# Interstitial Pregnancy after *In Vitro* Fertilization and Embryo Transfer Following Bilateral Salpingectomy: Report of Two Cases and Literature Review

**Published:** 2012-06-19

**Authors:** Elisabetta Garavaglia, Lavinia Quaranta, Anna Redaelli, Gabriella Colombo, Federica Pasi, Massimo Candiani

**Affiliations:** 1 Department of Obstetrics and Gynecology , San Raffaele Scientific Institute, Milan, Italy; 2 Università Vita-Salute San Raffaele, Milan, Italy

**Keywords:** Ectopic Pregnancy, Bilateral Salpingectomy, *In Vitro* Fertilization/ Embryo Transfer

## Abstract

Ectopic pregnancy is defined as the implantation and development of an embryo outside the uterus. Its incidence has increased over the past two decades. We report two cases of interstitial pregnancy on a tubal stump following bilateral salpingectomy and *in vitro* fertilization (IVF) treatments. We emphasize the importance of total salpingectomy during surgery in order to avoid interstitial pregnancy, particularly in women undergoing IVF treatments.

## Introduction

Ectopic pregnancy is defined as the implantation and development of an embryo outside the uterus. Its incidence has increased over the past two decades.This data is strongly associated with an increased incidence of pelvic inflammatory disease and of assisted reproductive technology (ART) with multiple embryo transfers.

Interstitial pregnancy is defined as implantation and development of an embryo in the proximal portion of the fallopian tubes. Its incidence ranges from 2 to 4% among ectopic pregnancies (1-3). Clinical manifestations include abdominal pain associated with vaginal bleeding (56-80%) and hypovolemic shock (2%). Diagnosis is based on ultrasound (US) (8-44%) and laparoscopy (45%).

Treatment guidelines have not yet been established. Interstitial pregnancy is associated with a maternal mortality rate of 2-3% compared to 0.14% for tubal ectopic pregnancy,which makes it an urgent and dangerous condition. Interstitial ectopic pregnancy can develop in a highly vascularized mass up to the second trimester before rupture, which may cause severe hemorrhage.

Here we report two cases of tubal stump pregnancies after bilateral salpingectomy and *in vitro* fertilization (IVF) treatments.

## Case Report 1

A 33-year-old woman (gravida 4, para0) with no history of pelvic disease had a history of an appendectomy in childhood and a diagnostic laparoscopy for an ovarian cyst in 2003. She experienced three ectopic pregnancies: the first ended in partial left salpingectomy in 2004; the second, located in the right tube,was treated with methotrexate (MTX) in 2006; and the third was followed by a right total laparoscopic salpingectomy in 2009.In August 2010, the patient was treated with IVF, but did not become pregnant.

In November 2010 two frozen embryos were transfered. On the 14^th^ day after embryo transfer, the serum beta-subunit of human chorionic gonadotrophin (beta-hCG) was 205 UI/mL; it rose to 732 UI/mL on the 16th day and 1633 UI/mL on the 19th day.

On the 22nd day after embryo transfer she was referred to our emergency department with complaints of lower abdominal pain and vaginal bleeding. Her vital signs were stable and a physical examination revealed diffuse lower abdominal tenderness with no signs of peritoneal irritation. Her hemoglobin level was 11.8 mg/dL. A transvaginal ultrasound (TVUS) revealed no intrauterine pregnancy sac and only a small accumulation of fluid in the Pouch of Douglas;her beta-hCG level was 1518 UI/mL. The patient was admitted to our gynecology ward and underwent TVUS and beta-hCG analyses every two days.

The day after admission to our ward a TVUS showed an accumulation of fluid in the cul-de-sac of 8.2x2.9 cm, and again no intrauterine pregnancy sac was detected in the uterine cavity. Serum hCG level splateaued as follows after embryo transfer: 2065 UI/mL (24<sup>th</sup> day), 2018 UI/mL (25th day), 1914 UI/mL (26<sup>th</sup> day), 1901 UI/mL (27<sup>th</sup> day), 2063 UI/mL (28<sup>th</sup> day), and 2173 UI/mL (29<sup>th</sup> day).

**Fig 1:**
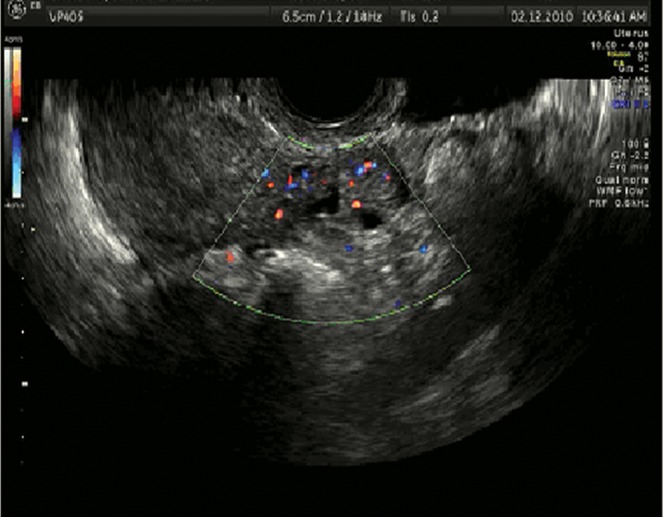
Cornual pregnancy with peripheral vascularization.

**Fig 2:**
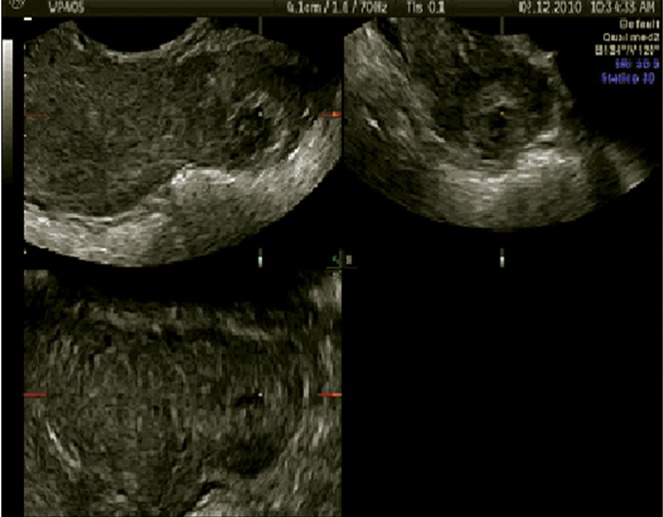
Left cornual pregnancy in sagittal, transversal, and coronal planes.

Finally, one month after embryo transfer TVUS showed a 25 mm mass in the left tubal angle apparently outside the myometrium,with no increase in the amount of free fluid in the cul-de-sac (100 mL; Figs 1-2). The next day the patient underwent laparoscopic resection of the left tubal stump (salpingectomy with cornuostomy).

Pathologic examination of the excised tubal stump revealed trophoblastic tissue.

## Case Report 2

A 37-year-old woman with a history of bilateral laparoscopic salpingectomy for bilateralhydrosalpinx in 2004 underwent IVF; two embryos were transfered in April 2005. After 10 days, serum betra- HCG was 61UI/mL, which increased to231UI/ mL after 12 days, and 1408 UI/mL after 17 days.

One month after embryo transfer the patient was referred to our emergency department with severe abdominal pain and an episode of vomiting. Physical examination revealed stablevital signs, a painful abdomen, positive Blumberg’s sign, and ahemoglobin of 8.6 mg/dL. TVUS revealed a large amount of free fluid and blood clots in the left abdominal quadrant, a complex irregular mass of 5-6 cm in maximum diameter was also revealed in the right tubal stump.The patient underwent a laparoscopic right cornuostomy. The pathologic examination of the excised tubal stump revealed trophoblastic tissue.

## Discussion

The prevalence of ectopic pregnancy ranges from 6 to 16% in the general population. The overall incidence has increased dramatically in the last two decades due to an increase in pelvic inflammatory disease and the introduction of medical assisted procreation techniques.

Other risk factors are: previous ectopic pregnancy (15%), tubal diseases and surgery, Diethylstilbestrol (DES) exposure during pregnancy, intrauterine contraception, infertility, multiple sexual partners, and smoking.

Recently IVF treatments have become more frequent due to increased maternal age at first pregnancy. ART represent an independent risk factor for ectopic and heterotopic pregnancies. The rate of heterotopic pregnancy (the simultaneous occurrence of intrauterine and ectopic gestation) after IVF is estimated to be as high as 0.3-1% (4).

Almost all ectopic pregnancies occur in the fallopian tube (95%), with the distribution of sites being: ampullary (76.9%), isthmic (12%), and fimbrial (11.1%). Ectopic pregnancies are ovarian (2%), interstitial or corneal (2%), and the remaining are abdominal or cervical (5).

Risk factors for interstitial pregnancies are similar to those for other tubal pregnancies (6), particularly salpingectomy (7-10). IVF treatments are also strongly associated with interstitial pregnancies (26.9%) (11-13).

The occurrence of interstitial pregnancy is estimated to be 1:3600 for all pregnancies achieved with IVF treatments. These pregnancies tend to be diagnosed later than most other ectopic pregnancies, as interstitial pregnancies can grow larger because the surrounding myometrium is more expandable than within the fallopian tube. Consequently, interstitial pregnancies have an increased risk of rupture, and early diagnosis is very important. In Bouyer’s study, almost one third of cornual ectopic pregnancies have been diagnosed after rupture with a significant hemoperitoneum. The typical rupture of these ectopic pregnancies occurs after 9 weeks and as late as 20 weeks (5).

Diagnosis of interstitial pregnancy is quite difficult and based upon clinical findings, imaging studies (ultrasound), and laboratory tests (hCG).

A TVUS is the most useful test in determining the location of an eectopic pregnancy, despite some studies that describe the use of MRI in diagnosing a pregnancy in a rudimentary horn (14, 15). However, imaging studies might not be diagnostic, as gestation is too early to be visualized. In this setting, the combination of TVUS and hCG can achieve diagnosis in almost 70% of cases (12, 16).

TVUS is very important for differential diagnosis among ectopic pregnancy sites.US diagnosis of cervical pregnancy requires the following criteria: enlargement of the cervix and uterus, diffuse amorphous intrauterine echoes, and no intrauterine pregnancy. The pregnancy sac must be below the internal cervical os, the cervical canal must be dilated, and the cervix must have a barrel shape (17).

US findings are useful to diagnose if an ovarian pregnancy can be a walled cystic mass within or adjacent to an ovary; however, Doppler US can not reliably distinguish between an ovarian implantation and a corpus luteum.

Three TVUS criteria are needed to diagnose an abdominal pregnancy, according to Studdiford in 1942: first, the absence of pathologic findings in the fallopian tubes; second, the absence of any uteroperitonel fistula; third, a pregnancy related to the peritoneal surface must be present to eliminate the possibility of a secondary implantation (18). However, the diagnosis of this type of EP is frequently made at the time of surgical intervention.

It can be difficult to differentiate between a spontaneous abortion in progress, cervico-isthmic pregnancy, and implantation within a cesarean scar (19). To diagnose this type of ectopic pregnancy, the TVUS features required are: an empty uterine cavity, an empty cervical canal, and development of the gestational sac in the anterior part of the uterine isthmus (20).

US findings highly suggesting an interstitial ectopic pregnancy are: the identification of an echogenic line between the gestational sac and the endometrial cavity and an empty uterine cavity with a gestational sac located outside the endometrial cavity with a myometrium less than 5 mm thick (21). If US imaging is equivocal, an MRI can be used. MRI criteria are the same as the TVUS criteria mentioned above (22).

It is very important to obtain an accurate medical history in order to identify women at risk of interstitial pregnancy, i.e. those who have undergone previous salpingectomy and a recent IVF.

This article underlines the importance of total salpingectomy and accurate cauterization of the tubal stump in patients undergoing surgery for ectopic pregnancy in the fallopian tube in order to avoid the risk of a consequent interstitial pregnancy. Particular attention must be taken in women undergoing IVF treatments.

Uterine rupture in pregnancies following salpingectomy for corneal pregnancy has been described (23). On the contrary, in Ng’s experience, no cases of uterine rupture occurred in 18 (34%) women previously treated with surgery and who became pregnant, 10 of who marrived at term (16). The same observation was reported by Moon (24).

Management of interstitial pregnancy varies widely in the literature (16, 25-27), and primary treatment can be surgical or medical. Surgery can be performed by laparoscopy or laparotomy and can be radical (hysterectomy) or conservative (cornuostomy or cornual resection) (25). Cornual pregnancy has also been treated by hysteroscopy (28). Patients with interstitial pregnancy at an early stage are candidates for medical treatment that consists of single or multiple dose MTX (29). According to Larrain, no failures have been noted among patients who received combined primary treatment with surgery and administration of MTX (25).

Both of our patients were treated with surgery; the second patient particularly underwent surgery in order to avoid a second ectopic pregnancy in the same position after further IVF treatment.

In conclusion, after IVF treatment, and particularly in patients with prior bilateral salpingectomy, special attention to interstitial pregnancy is warranted, as it remains a life-threatening condition. Surgery remains the mainstay treatment among patients who have undergone a previous partial salpingectomy.
